# Supplementary figures and images for: Identification of a Novel Eight-Gene Risk Model for Predicting Survival in Glioblastoma: A Comprehensive Bioinformatic Analysis
Source: Cancers (Basel). 2023 Jul 31;15(15):3899. doi: 10.3390/cancers15153899 (PMC10417140; doi:10.3390/cancers15153899)

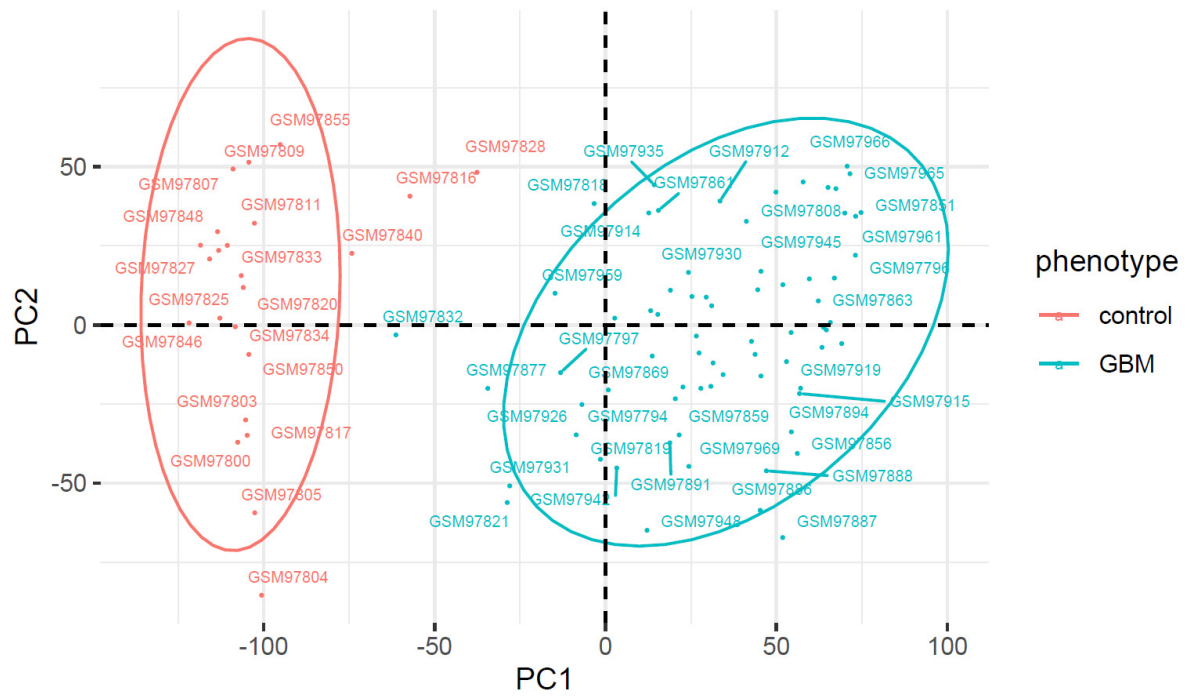

Figure S1: PCA results of the GSE4290 dataset.

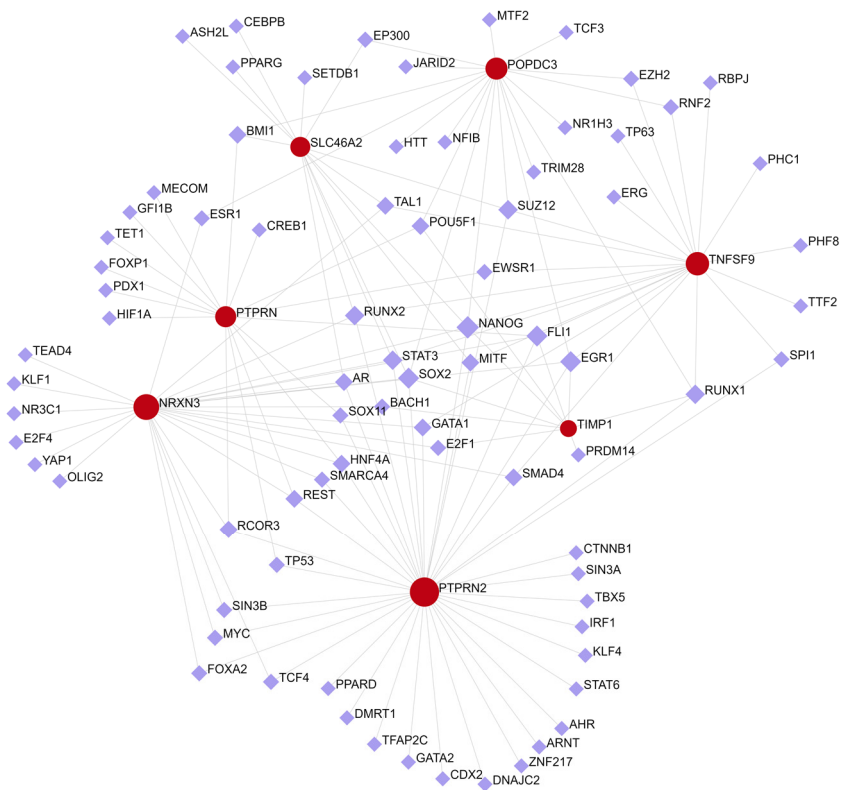

Figure S2: Network analysis of the eight-gene signature.

Supplement: Supplementary file 1 [file cancers-15-03899-s001.zip › cancers-2467678-Supplementary Figures.pdf]
